# Vascular Dysfunction as Target Organ Damage in Animal Models of Hypertension

**DOI:** 10.1155/2012/187526

**Published:** 2012-02-14

**Authors:** Mario Fritsch Neves, Daniel Arthur B. Kasal, Ana Rosa Cunha, Fernanda Medeiros

**Affiliations:** ^1^Department of Clinical Medicine, State University of Rio de Janeiro, Avenue 28 de Setembro, 77 sala 329, 20551-030 Rio de Janeiro, RJ, Brazil; ^2^Faculty of Nutrition, Federal University of Rio de Janeiro, Rua Mariz e Barros 775, 20270-004 Rio de Janeiro, RJ, Brazil

## Abstract

Endothelial dysfunction is one of the main characteristics of chronic hypertension and it is characterized by impaired nitric oxide (NO) bioactivity determined by increased levels of reactive oxygen species. Endothelial function is usually evaluated by measuring the vasodilation induced by the local NO production stimulated by external mechanical or pharmacological agent. These vascular reactivity tests may be carried out in different models of experimental hypertension such as NO-deficient rats, spontaneously hypertensive rats, salt-sensitive rats, and many others. Wire myograph and pressurized myograph are the principal methods used for vascular studies. Usually, increasing concentrations of the vasodilator acetylcholine are added in cumulative manner to perform endothelium-dependent concentration-response curves. Analysis of vascular mechanics is relevant to identify arterial stiffness. Both endothelial dysfunction and vascular stiffness have been shown to be associated with increased cardiovascular risk.

## 1. Introduction

Hypertension contributes significantly to global cardiovascular morbidity and mortality. It appears to have a multifaceted connection with endothelial dysfunction that arises before the development of adverse cardiovascular events [1]. Hypertension is associated with endothelial dysfunction in the peripheral, coronary, and renal circulations in the majority of studies, indicating an important mechanism whereby hypertension promotes the development of vascular disease [1–4].

The endothelium is currently recognized to be essential for the regulation of the vascular tone and structure [5]. Functional and structural vascular changes can develop in the presence of cardiovascular risk factors, enhancing the process of atherosclerosis. In this situation, there is a imbalance between endothelium-derived nitric oxide (NO) and other vasoprotective factors. Moreover, an excessive production of proinflammatory and vasoconstrictors substances such as angiotensin II, endothelin 1 (ET-1), and reactive oxygen species (ROS) may contribute to impairment of endothelial function, increasing the peripheral vascular resistance which contributes to blood pressure raise and cardiovascular remodeling [6]. In fact, endothelial dysfunction is one of the main characteristics of chronic hypertension, and it is characterized by impaired NO bioactivity determined by increased levels of ROS [7]. Other systems contribute to this imbalance such as renin-angiotensin and ET-1 which may participate in the pathogenesis of endothelial dysfunction. Indeed, ET-1 activity is increased along with reduced bioavailability of NO [8].

Endothelial dysfunction is not specific to primary hypertension since it may be associated with other risk factors and cardiovascular diseases. Furthermore, there is no correlation between the severity of endothelial dysfunction and blood pressure levels. These two considerations indicate that blood pressure raise is not determined by impairment of endothelial function. On the other side, it has been reported that an impaired response to acetylcholine correlates with carotid intima-media thickening in patients with essential hypertension [9]. The presence of coronary endothelial dysfunction has also been associated with cardiovascular events in longitudinal studies [10]. Therefore, further studies are required to ascertain a direct relationship between endothelial dysfunction and cardiovascular events in patients with essential hypertension.

## 2. Vascular Changes Induced by Hypertension

Primary hypertension is characterized by an increase in peripheral vascular resistance. In animal models of hypertension small arteries (lumen diameter between 100 and 400 *μ*m) develop lesions induced by hypertension, and can be subjected to *ex vivo* studies [11]. The main determinant of an increase in peripheral vascular resistance is the reduction in lumen diameter. According to Poiseuille's law, resistance varies inversely with the fourth power of the blood vessel radius. As a consequence, small reductions in vascular lumen increase arterial resistance into a considerable extent [12].

 Three important vascular components are affected by hypertension: vascular structure, vascular mechanics (stiffness), and vascular function [13]. Structural modifications involve the reduction of lumen diameter and the thickening of the vascular media, causing an increase in the media/lumen ratio. The increase in media/lumen ratio can result from a reduction of external diameter, causing a narrowing of the lumen without an increase in total diameter (eutrophic remodelling) or can occur due to media thickening (hypertrophic remodeling) [14].

 Hypertension can also determine modifications in the mechanical properties of an artery, promoting an increase in vascular stiffness. These modifications can involve the expression and localization of extracellular matrix components, such as collagen and integrins [15]. The hyperactivity of the renin-angiotensin system is the main trigger for these alterations. In addition, it has been previously demonstrated that angiotensin II-induced vascular changes were at least partially prevented by aldosterone antagonism, indicating that some vascular actions of angiotensin II may be mediated by aldosterone [16]. On the other hand, functional impairment reflects a reduction in vasodilation induced by nitric oxide, also called endothelium-dependent response, measured by the acetylcholine response curve [17].

## 3. Pathophysiology of Endothelial Dysfunction

NO is one of the most important vasodilating substances released by the endothelium, acting as a vasodilator and inhibiting growth and inflammation. Impaired endothelial function has been directly associated with decreased bioavailability of NO which can be originated mainly from oxidative stress and inflammation [18]. In fact, C-reactive protein has been shown to diminish endothelial NO synthase (eNOS) [19], the enzyme that produces NO using L-arginine as substrate. Moreover, both processes are connected since reactive oxygen species (ROS) are able to upregulate intercellular adhesion molecule1 (ICAM-1), vascular adhesion molecule1 (VCAM-1), and macrophage chemoattractant peptide1 (MCP-1) [20]. In human hypertension and in animal models of hypertension, NAD(P)H oxidase has been demonstrated to be the main source for oxidative excess, although other sources, such as xanthine oxidase and mitochondria, have also been described [21, 22]. In experimental models of hypertension with hyperactivity of renin-angiotensin system, such as angiotensin II-infused rats, increased ROS are stimulated by NAD(P)H oxidase, resulting in vascular inflammation [23]. Animal models of hyperhomocysteinemia have been associated with endothelial dysfunction even with no elevation of blood pressure [24]. Mild hyperhomocysteinemia has also been linked to stiffer small arteries with increased collagen deposition in the vascular wall. These alterations may be stressed by angiotensin II-induced hypertension [25]. This finding may explain the increased cardiovascular risk linked to hyperhomocysteinemia.

It is well known that NO is a volatile substance, with a very short half-life. Therefore, its bioavailability is usually evaluated by measuring the vasodilation induced by the local NO production stimulated by specific external mechanical and pharmacological agent, that is, through vascular reactivity tests [26].

## 4. Relationship between Macro- and Microcirculation

The main purpose of large arteries is to transport a satisfactory blood supply from the heart to peripheral tissues. In addition to conduit function, large arteries have a cushioning function that consists to transform the pulsatile flow of arterial vessels into the steady flow required for oxygen supply. Interactions between macro- and microvascular changes seem to contribute to end-organ damage in hypertension. The properties of conduit arteries are affected by hypertension resulting in reduced arterial compliance and distensibility. At the microvascular level, hypertensive disease is characterized by eutrophic or hypertrophic remodeling and capillary rarefaction [27]. Central pulse pressure may be transmitted to microcirculation in circumstances where the autoregulatory mechanisms normally protecting vital organs are offset. In fact, a microvascular disease is commonly observed in association with end-organ damage [28].

After ventricular ejection, blood pressure propagates along the arterial tree as a wave. Mainly at arteriolar branching, this wave may be reflected and then becomes retrograde. In young individuals, the wave summation takes place in early diastole, thus improving the diastolic coronary perfusion without disturbing cardiac afterload. A stiffer aorta implicates in greater pulse wave velocity, causing the reflections to occur in late systole rather than in diastole as in healthy subjects. Under this condition, coronary perfusion is impaired, leading to myocardial ischemia [29].

It has been hypothesized that increased arterial stiffness of the large arteries may lead to microvascular changes due to increased pulsatile flow. Pulse pressure and mean arterial pressure (MAP) have been considered risk factors for cardiovascular disease. MAP is the steady component of the blood pressure curve and it is determined by cardiac output and microvascular resistance. On the other hand, pulse pressure is the pulsatile component that is determined by left ventricular ejection, the compliance of the large arteries, and the intensity of wave reflections from the microcirculation [30].

The relationship between endothelial function and vascular stiffness is still controversial. Witte et al. showed that forearm flow-mediated dilation (FMD) responses were affected by arterial stiffness [31]. In addition, Parvathaneni et al. showed a linear correlation between FMD responses and both small and large arterial stiffness [32]. On the other hand, neither large nor small arterial stiffness was considered viable indicators of endothelial or microcirculatory reactivity in healthy young males [33].

## 5. Principal Models of Experimental Hypertension

Chronic inhibition of NO synthase by N^G^-nitro-L-arginine methyl ester (L-NAME) in adult rats produces endothelial dysfunction with increase of vascular responsiveness to adrenergic stimuli associated with perivascular inflammation [27]. Vascular remodeling, renin-angiotensin system, sympathetic nervous system, and endothelium-derived constricting factors (EDCF) also play a role in L-NAME-induced hypertension [28, 29]. Obviously, reduced NO synthesis is the main mechanism for the blood pressure elevation induced by L-NAME. Interestingly, continuation of L-NAME administration from 4 to 8 weeks further increases blood pressure, but not enhancing the impairment of endothelium-dependent relaxation. It has been recently reported that, after 4 weeks of L-NAME treatment, endothelial NO synthase (eNOS) expression in the heart was significantly increased and this increase was amplified after 7 weeks of treatment [30]. This finding suggests that the upregulation of eNOS protein expression represents one of counterregulatory mechanisms activated to compensate the blood pressure raise. Impairment of NO signaling has been improved after the cessation of L-NAME administration. However, persisting arterial structural alterations and enhanced EDCF formation may decelerate blood pressure reduction even after the restoration of NO synthase activity [29].

 Spontaneously hypertensive rats (SHR) seem to be the experimental model most similar to the human primary hypertension. The involvement of NO for blood pressure elevation in these animals is still controversial. Notably, endothelium-dependent dilation is preserved in the prehypertensive and early hypertensive stages of SHR [31]. Moreover, impaired endothelium-dependent relaxation was not found in rats with borderline hypertension. This has been supported by the finding that their aortic NO production was even elevated in comparison with normotensive rats [32].

 The increased sympathetic activity contributes to endothelial dysfunction and blood pressure elevation in SHR. It has been shown that NO counterbalances angiotensin II effects on sympathetic stimulation [33]. It has been recently demonstrated that inhibition of angiotensin converting enzyme lowers blood pressure in SHR by attenuating sympathetic tone [34].

 The cause for impairment of endothelial function in hypertension is not completely elucidated. When comparing SHR with NO-deficient rats, it has been shown that alterations in the vascular wall in both groups of animals but the endothelium-dependent dilation in aorta is clearly reduced in NO-deficient rats while relatively preserved in SHR [35]. This finding indicates that endothelial dysfunction in hypertension is not related to vascular structural changes.

 In salt-dependent hypertension, the reduction of NO bioavailability caused by increased ROS levels seems to play a significant role in the pathogenesis of blood pressure elevation and endothelial dysfunction [36]. Renal hemodynamics may be changed by local oxidative stress resulting in sodium retention [37]. Particularly, renin-angiotensin system does not contribute to hypertension in this experimental model. On the other hand, sympathetic hyperactivity has been shown in salt-hypertensive Dahl rats leading to vasoconstriction which becomes superior to NO-dependent vasodilation [38].

## 6. Vascular Reactivity by Wire Myograph

In some experimental studies, an artery can be dissected, immediately immersed in cold physiological salt solution (PSS), and cleaned of adipose or connective tissue to be mounted as ring-shaped preparations in the small vessel wire myograph. Two 40 *μ*m stainless steel wires are passed through the lumen of the vessel, and mounted in the jaws of the wire myograph. After 30 min equilibration in oxygenated (5% CO_2_, 95% O_2_ mixture) PSS, a standardized computer-assisted normalization procedure is performed to set the pre-tension of the arteries. This defines the lumen diameter that the artery would have had *in vivo* when relaxed and under a transmural pressure of 100 mmHg. The arteries were then set to the lumen diameter of 90% of the normalized inner diameter when active force development was maximal [39].

 Before the start of measurements, the vessels are allowed to stabilize in PSS for 30 minutes. After preconstriction with noradrenaline addition, the arterial contraction reaches a steady state. Thus, increasing concentrations of the vasodilator acetylcholine are added in cumulative manner to perform endothelium-dependent concentration-response curves [40]. There are many agents, such as histamine, bradykinin, ATP, and serotonin, which produce arterial relaxation either by direct action on the smooth muscle cell or by indirect action mediated by activation of endothelial receptors. However, acetylcholine is the more commonly used substance to activate endothelial receptors in this setting.

## 7. Vascular Studies Using Pressurized Myography

### 7.1. Preparing Vessels for Functional Studies

**Figure 1:**
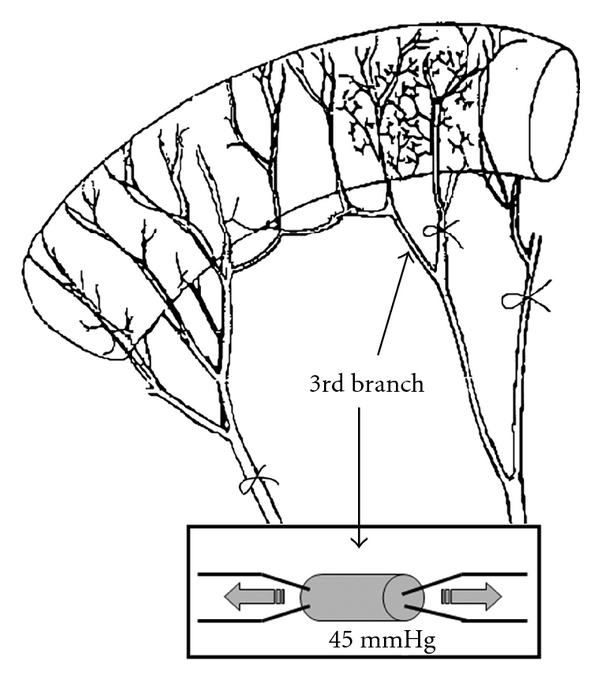
The third branch of mesenteric vessels to be dissected and mounted in a pressurized myograph in rat studies. The vessel is attached to micropipettes, connected to transducers keeping 45 mmHg as a fixed intraluminal pressure for functional studies.

A technique that provides important information about structural, mechanical, and functional properties in small vessels is the pressurized myography (Figure 1). This method offers advantages for the analysis of structural features compared to conventional histology techniques, because it observes the vessel in *ex vivo* preparation, without the artefacts induced by tissue fixation [41]. In the pressurized myograph, the vessel is dissected and cannulated in both ends with micropippetes that are connected to intraluminal pressure transducers, allowing pressure control. The perfusion chamber where the vessel is immersed is supplied with a perfusion system that keeps changing a physiological solution continuously. This allows the infusion of pharmaceuticals with different concentrations for the evaluation of vascular diameter, indicating its functional properties. The vessel image is obtained with the aid of optic microscopy, recorded by a video camera and exposed in a video monitor. This image is analysed for the determination of wall thickness and vascular diameter [42, 43].

### 7.2. Evaluation of Structural Changes

From data obtained with the pressurized myograph, we can gather information about arterial structure, employing formulas that take into account the cylindrical geometry of the vessel [44]. As a result, when we apply to the vessel a constant intraluminal pressure of 45 mmHg, the media cross-sectional area (CSA) is obtained subtracting the external from the internal vascular transversal area:

(1) $$\text{CSA}=\left[\left(\frac{\pi }{4}\right)\left(D_{e}^{2}\;-\;D_{i}^{2}\right)\right],$$

where *D*_*e*_ and *D*_*i*_ are the external and internal diameters, respectively. It is also possible to adjust intraluminal pressure, in order to observe the variations in the external diameter of the vessel, which correspond to the extent of vascular stiffness.

### 7.3. Vascular Mechanics

In vascular mechanics evaluation, resistance arteries must be previously deactivated by extraluminal perfusion with calcium-free PSS for 30 min in order to eliminate any myogenic tone. Intraluminal pressure is then increased to 140 mmHg three times, and the cannula must be adjusted until the artery walls are straight and parallel to each other. A servo-controlled pump is used to increase intraluminal pressure in increments of 10 mmHg until a pressure of 40 mmHg is achieved, and then in 20 mmHg increments until a pressure of 140 mmHg is reached. Media thickness and lumen diameter must be assessed at each pressure level at different points along the vessel, and the average is used in subsequent calculations. The initial diameter is measured at 3 mmHg unless the vessel collapses. In these cases, lumen diameter has to be estimated by fitting the intraluminal pressure-lumen diameter data with a third-order polynomial equation. The incremental distensibility is the percentage of change in lumen diameter (Δ*D*/*D*) for a given change in intraluminal pressure (Δ*P*):

(2) $$\text{Incremental}\;\text{distensibility}=\left(\frac{1}{\Delta P}\right)\times \left(\frac{\Delta D}{D}\right)\times 100.$$

Circunferencial strain (*ε*) is calculated as *ε* = (*D* − *D*_0_)/*D*_0_, where *D* is the lumen diameter observed in a given intraluminal pressure and *D*_0_ corresponds to the original diameter or estimated with intraluminal pressure of 3 mmHg. The circumferential stress (*σ*) is calculated as *σ* = (*PD*)/2*M*, where *P* is intraluminal pressure, *D* and *M* are luminal diameter and media thickness, respectively. Pressure is converted from mmHg to dyn/cm^2^ (1 mmHg = 1,334 × 10^3^ dyn/cm^2^).

## 8. Conclusions

In conclusion, although not specific to hypertension, endothelial dysfunction frequently develops in hypertensive animals. The reduction of NO bioavailability caused by increased ROS is the main mechanism involved in the pathogenesis of endothelial dysfunction. Nevertheless, hyperactivity of renin-angiotensin system, endothelin, sympathetic activation, and inflammation may also contribute to oxidative stress and endothelial dysfunction. The participation of these systems may vary in different animal models of hypertension. Wire myograph and pressurized myograph are the key methods for vascular evaluation in the experimental setting. Along with endothelial function evaluation, analysis of vascular mechanics is relevant to identify arterial stiffness which appears to be associated with cardiovascular events.
